# The effects of leptin and cannabinoid CB1 receptor agonist/antagonist in cerebral tissues of epileptic rats

**DOI:** 10.1590/1806-9282.20231333

**Published:** 2024-05-20

**Authors:** Mesut Kılıçoğlu, Uğur Düz, Gökhan Arslan, Mustafa Ayyıldız, Erdal Ağar, Nermin Kılıç

**Affiliations:** 1 Kayseri Education and Research Hospital, Department of Clinical Biochemistry, Kayseri, Türkiye; 2 Aydın Provincial Directorate of Health, Public Health Laboratory, Aydın, Türkiye; 3 Department of Physiology, Faculty of Medicine, University of Ondokuz Mayıs, Samsun, Türkiye; 4 Department of Clinical Biochemistry, Faculty of Medicine, University of Ondokuz Mayıs, Samsun, Türkiye

**Keywords:** Epilepsy, Penicillin, Leptin, Cannabinoids, ACEA, AM251

## Abstract

**OBJECTIVE:**

In this study, the effects of leptin, cannabinoid-1 (CB1) receptor agonist ACEA and antagonist AM251, and the interactions between leptin and CB1 receptor agonist/antagonist on oxidant and antioxidant enzymes in the cerebrum, cerebellum, and pedunculus cerebri tissue samples were investigated in the penicillin-induced epileptic model.

**METHODS:**

Male Wistar albino rats (n=56) were included in this study. In anesthetized animals, 500 IU penicillin-G potassium was injected into the cortex to induce epileptiform activity. Leptin (1 μg), ACEA (7.5 μg), AM251 (0.25 μg), and the combinations of the leptin+ACEA and leptin+AM251 were administered intracerebroventricularly (i.c.v.) after penicillin injections. Malondialdehyde (MDA), superoxide dismutase (SOD), and glutathione peroxidase (GPx) levels were measured in the cerebral tissue samples and plasma with the ELISA method.

**RESULTS:**

MDA levels increased, while SOD and GPx levels decreased after penicillin injection in the cerebrum and cerebellum. The efficacy of penicillin on SOD, MDA and GPx levels was further enhanced after leptin or AM251 injections. Whereas, ACEA decreased the MDA levels and increased GPx levels compared with the penicillin group. Administration of AM251+leptin did not change any oxidation parameter compared with the AM251. Furthermore, co-administration of ACEA and leptin significantly increased oxidative stress compared with the ACEA-treated group by increasing MDA and decreasing GPx levels.

**CONCLUSION:**

It was concluded that leptin reversed the effect of ACEA on oxidative stress. Co-administration of AM251 and leptin did not change oxidative stress compared with the AM251-treated group suggesting AM251 and leptin affect oxidative stress using the same pathways.

## INTRODUCTION

Epilepsy is a disorder characterized by the consequences of cognitive, psychological, neurobiological and social status and a predisposition to produce continuous epileptic seizures. The limitations and difficulties of epilepsy research have led researchers to epileptic animal models. In vivo and in vitro studies have been performed with many substances (pentylenetetrazole, bicuculline, picrotoxin, penicillin, etc.) to reveal the molecular mechanism of seizure activity in experimental epilepsy models^1-3^.

Reactive oxygen radicals (ROS) including malondialdehyde (MDA) and superoxide dismutase (SOD) are thought to play important roles in epilepsy formation and neuronal death following seizures^4^. Many studies using penicillin, kainate, pilocarpine and PTZ to induce epileptic seizures have shown that oxidative stress may be involved in the pathophysiology of epilepsy^5^.

The main role of leptin in the body is to regulate food intake and energy metabolism with a negative feedback effect on the cerebrum (especially the hypothalamus) and to prevent the development of obesity^6^. Leptin has also been found to increase nerve cell excitability in various studies^3,7^. Leptin has been shown to increase neuronal excitability by increasing NMDA receptor and synaptic transmission in rat hippocampal cell culture and cerebrum slices by increasing intracellular calcium^8^. In another rat study, leptin increased action potentials in electrophysiological recordings from proopiomelanocortin-type neurons^7^.

Cannabinoids are chemical substances obtained from a plant called Cannabis sativa. Cannabinoids, whose anticonvulsant effects have been known for centuries, are known to produce anticonvulsant effects via CB1 receptors in many experimental epilepsy models^3,9,10^. Recent studies on the functions of leptin suggest that there may be a relationship between leptin and cannabinoids^11,12^. In obese Zucker rats, it was revealed that leptin regulates the eating function via cannabinoid CB1 receptors in the subcortical and limbic areas of the cerebrum^11^. In addition, CB1 receptor mRNA levels showed steady increases in obese Zucker rats. This indicates that the signaling of leptin on CB1 receptor mRNA is impaired^11^.

In the literature, there is no study showing the effects of combined administration of leptin and CB1 receptor agonist/antagonist on oxidative stress parameters. Therefore, we aimed to investigate these combined effects.

## METHODS

All experimental procedures were approved by the Institutional Animal Care and Use Committee of the Ondokuz Mayis University (2009/61) and were conducted in accordance with the ARRIVE guidelines and the Guide for the Care and Use of Laboratory Animals as per the US National Institutes of Health (NIH Publications No. 8023, revised 1978). In the experiments, 56 male Albino Wistar were divided into 8 groups with 7 animals in each group. Rats were purchased from the Animal House of Ondokuz Mayis University, Samsun, Türkiye. The rats were maintained in a temperature (22 ± 10C) and humidity-controlled (55% ± 5) room on a 12-hour light/dark cycle. All animals were given free access to standard rat food and tap water ad libitum.

### Experimental Groups

Experimental procedures for ECoG recordings and injections are described in detail in the study Arslan et al.^3^ and the tissues were used in this study. In anesthetized animals, 500 IU Penicillin-G potassium was injected into the cortex and the control group was given sterile physiological saline (SF). The most effective dose of leptin on epileptiform activity was determined based on the data of previous study^3^. CB1 receptor agonist ACEA 7.5 μg (i.c.v.) and CB1 receptor antagonist AM251 0.25 μg (i.c.v.) were used to investigate the effects of the cannabinoid system on epileptic activity^3^. Then, cannabinoid receptor agonist and antagonist were combined with leptin to examine the interaction between these pathways.

### Biochemical Analyses

After the ECoG recordings^3^, intracardiac 6-8 ml of blood was collected from the rats and the blood was transferred into EDTA tubes and biochemistry tubes without anticoagulant. The blood was centrifuged and the plasma was separated. After reperfusion with saline (0.9% NaCl), cerebrum, cerebellum and pedunculus cerebri tissues were removed. The tissues were frozen with liquid nitrogen and stored at -80°C until the day of our study.

MDA, SOD and GPx levels were measured in the plasma and the tissue samples. Protein determination in tissue was assessed by a commercial enzyme-linked immunosorbent assay kit (Biotek, USA) using the Lowry method^13^. Plasma and tissue malondialdehyde-lipid peroxidation levels were measured spectrophotometrically by the thiobarbituric acid-reactive substrates (TBARS) method. Plasma and tissue glutathione peroxidase levels and plasma SOD levels were measured by the ELISA method following the manufacturer's kit instructions (CAYMAN Chemical, Ann Arbor, MI, USA). Results were calculated per mg.protein.

### Statistical analysis

The data obtained from the study were analyzed in SPSS 17.0 package program. Shapiro-Wilk test was performed whether the data was normally distributed. After it was determined that the data did not fit the normal distribution, Kruskal-Wallis test was used for intergroup comparisons. Statistical significance level was accepted as p<0.05. Descriptive characteristics of the data were expressed as mean and median (minimum-maximum).

## RESULTS

MDA, which is an indicator of oxidative damage, and GPx and SOD, which are antioxidant enzymes, were measured and statistically evaluated in the cerebrum, cerebellum, pedunculus cerebri tissues and plasma samples.

### MDA levels in the cerebral tissues and plasma

In the cerebrum tissue; MDA levels were significantly higher in the penicillin group compared with the control group (p<0.01), higher in the penicillin group compared with the leptin group (p<0.05), higher in the penicillin+leptin group compared with the penicillin group (p<0.01), higher in the penicillin+AM251 group compared with the penicillin group (p<0.01), lower in penicillin+ACEA group compared with the penicillin group (p<0.01), and higher in the penicillin+ACEA+leptin group compared with the penicillin+ACEA group (p<0.01) (Table 1).

**Table 1:** Tissue malondialdehyde levels of the study groups

Groups	MDA Cerebrum (μmol/g)	MDA Cerebellum (μmol/g)	MDA Ped. Cerebri (μmol/g)
Control (SF)	2.3 (1.6-4.3)	3.8 (3.2-6.8)	2.6 (1.6-2.8)
Penicillin	4.5 (2.8-11.7)^a^	5.4 (4.1-13.5)^aa^	2.6 (2.3-4.3)
Leptin	3.5 (1.8-4.0)^b^	4.6 (3.3-5.7)^bb^	2.7 (2.1-3.4)
Penicillin + leptin	8.7 (6.8-15.6)^c^	8.8 (6.0-13.6)^cc^	2.6 (2.3-4.8)
Penicillin + AM251	7.7 (7.2-9.1)^d^	9.3 (8.3-9.9)^dd^	2.8 (2.5-27.5)
Penicillin + ACEA	2.5 (1.8-3.2)^e^	4.2 (3.1-5.4)	3.3 (2.5-3.5)
Penicillin + AM251 + leptin	9.8 (5.9-11.0)	11.0 (6.2-13.8)	2.6 (1.8-3.3)
Penicillin + ACEA + leptin	5.6 (4.0-7.1)^f^	7.7 (4.5-9.8)^ff^	3.0 (1.6-15.6)

Kruskal-Wallis test was used. Values are given as median (min-max).

a p< 0.01,

aa p< 0.05; between control and penicillin groups;

b p< 0.05,

bb p< 0.05; between penicillin and leptin groups;

c p< 0.01,

cc p< 0.05; between penicillin and penicillin+leptin groups;

d p< 0.01,

dd p< 0.01; between penicillin and penicillin+AM251 groups;

e p< 0.01; between penicillin and penicillin+ACEA groups;

f p< 0.01,

ff p< 0.01; between penicillin+ACEA and penicillin+ACEA+leptin groups.

MDA levels in the cerebellum tissue were significantly higher in the penicillin group compared with the control group (p<0.05), higher in the penicillin group compared with the leptin group (p<0.05), higher in the penicillin+leptin group compared with the penicillin group (p<0. 05), higher in the penicillin+AM251 group compared with the penicillin group (p<0.01), lower in penicillin+ACEA group compared with the penicillin group (p<0.05), and higher in the penicillin+ACEA+leptin group compared with the penicillin+ACEA group (p<0.01) (Table 1).

No significant difference was found in MDA levels among any group in the pedunculus cerebri tissue and plasma (p>0.05).

### SOD levels in the cerebral tissues and plasma

In cerebrum tissue; SOD levels were significantly lower in the penicillin group compared with the control group (p<0.01), lower in the penicillin group compared with the leptin group (p<0.01), lower in the penicillin+leptin group compared with the penicillin group (p<0.05), lower in the penicillin+AM251 group compared with the penicillin group (p<0.01) (Table 2).

**Table 2:** Tissue superoxide dismutase levels of the study groups.

Groups	SOD Cerebrum (U/mg)	SOD Cerebellum (U/mg)	SOD Ped. Cerebri (U/mg)
Control (SF)	324 (287-433)	186 (135-214)	156 (68-292)
Penicillin	182 (143-209)^a^	102 (47-153)^aa^	158 (96-224)
Leptin	282 (254-392)^b^	153 (109-199)^bb^	163 (64-309)
Penicillin + leptin	74 (49-93)^c^	71 (44-90)	122 (111-248)
Penicillin + AM251	82 (27-101)^d^	58 (30-67)^dd^	131 (38-202)
Penicillin + ACEA	169 (86-347)	110 (47-413)	161 (69-309)
Penicillin + AM251 + leptin	38 (26-98)	61 (49-74)	148 (103-201)
Penicillin + ACEA + leptin	122 (56-308)	68 (29-155)	115 (68-258)

Kruskal-Wallis test was used. Values are given as median (min-max).

a p< 0.01,

aa p< 0.01; between control and penicillin groups;

b p< 0.01,

bb p< 0.05; between penicillin and leptin groups;

c p< 0.05; between penicillin and penicillin+leptin groups;

d p< 0.01

dd p< 0.05; between penicillin and penicillin+AM251 groups.

In the cerebellum tissues; SOD levels were significantly lower in the penicillin group compared with the control group (p<0.01), lower in the penicillin group compared with the leptin group (p<0.05), lower in the penicillin+AM251 group compared with the penicillin group (p<0.05)

There were no significant differences among any groups in SOD levels in the pedunculus cerebri tissue and plasma.

### GPx levels in the cerebral tissues and plasma

In cerebrum tissue, GPx levels were significantly lower in the penicillin group compared with the control group (p<0.01), lower in the penicillin group compared with the leptin group (p<0.05), lower in the penicillin+leptin group compared with the penicillin group (p<0.05), higher in the penicillin+ACEA group compared with the penicillin group (p<0.05), and significantly lower in penicillin+ACEA+leptin group compared with the penicillin+ACEA group (p<0.01) (Table 3).

**Table 3:** Tissue glutathione peroxidase levels of the study groups

Groups	GPx Cerebrum (nmol/mg)	GPx Cerebellum (nmol/mg)	GPx Ped. Cerebri (nmol/mg)
Control (SF)	11.4 (9.4-15.5)	13.1 (8.5-23.7)	8.0 (4.2-18.3)
Penicillin	6.3 (4.4-7.6)^a^	6.6 (2.5-15.9)	8.6 (2.6-16.8)
Leptin	8.7 (6.0-15.3)^b^	12.2 (5.9-17.9)	8.3 (3.1-12.5)
Penicillin + leptin	4.2 (3.0-5.4)^c^	7.7 (2.4-9.0)	6.5 (2.2-17.6)
Penicillin + AM251	4.4 (2.5-8.8)	6.9 (2.7-13.3)	7.8 (1.9-9.6)
Penicillin + ACEA	8.9 (4.6-13.8)^d^	6.8 (2.4-17.0)	8.2 (2.3-14.9)
Penicillin + AM251 + leptin	2.0 (0.6-12.3)	6.2 (3.1-7.5)	6.2 (2.9-15.4)
Penicillin + ACEA + leptin	3.6 (0.6-12.5)^e^	6.2 (4.0-12.0)	7.0 (2.6-13.4)

Kruskal-Wallis test was used. Values are given as median (min-max).

a p< 0.01; between control and penicillin groups;

b p< 0.01; between penicillin and leptin groups;

c p< 0.05; between penicillin and penicillin+leptin groups;

d p< 0.05; between penicillin and penicillin+ACEA groups;

e p< 0.01; between penicillin+ACEA and penicillin+ACEA+leptin groups.

No significant difference was found in GPx levels among any group in the pedunculus cerebri tissue and plasma (p>0.05).

## DISCUSSION

Animal models of epilepsy are used to understand the pathophysiology of seizures and to develop new therapies to treat epilepsy^3,14^. Penicillin is one of the most widely used drugs for inducing experimental seizures. With the administration of intracortical penicillin, γ-aminobutyric acid (GABA) type-A receptors are inhibited and this suppresses the chlorine entry inside the neurons resulting in focal seizure focus which is recorded from the surface of the cortex via ECoG^2,3^.

Free radical production causes the accumulation of excitatory neurotransmitter glutamic acid and a decrease in GABA, an inhibitory neurotransmitter, in the cerebrum^14^. Free oxygen radicals are thought to play important roles in the formation of epilepsy and neuronal death following seizures^5,15^. Obay et al. declared that in the cerebrum, MDA levels were increased, while SOD and GSH levels were decreased after pentylenetetrazole-induced seizures in rats^15^. Parallel to this study, we found an increase in MDA levels and a decrease in GPx and SOD levels observed in the cerebrum and cerebellum tissues of rats after penicillin-induced epileptiform activity.

Leptin has been found to increase nerve cell excitability in some studies^3,7^. Arslan et al. reported that leptin increased epileptiform activity in the experimental epilepsy model induced by penicillin in rats^3^. Moreover, leptin increases excitability by increasing intracellular calcium and synaptic transmission^7^. Kutlu et al. reported that lipid peroxidase and glutathione levels were decreased in the cerebrum as a result of leptin administration^16^. Furthermore, the external application of leptin did not change MDA levels in a glial cell culture study^17^. We determined that the leptin administration to the non-epileptic animals did not have a significant effect on oxidative stress. However, leptin significantly increased the MDA levels in the cerebrum and cerebellum tissues of the rats compared to the penicillin group and significantly decreased the SOD and GPx levels suggesting that leptin may show its proconvulsant effect through oxidative stress probably by increasing intracellular Ca^+2^ levels.

Cannabinoids CB1 and CB2 receptors are G protein-coupled receptors and belong to different families of cell membrane-bound proteins. CB1 receptors inhibit presynaptic N and P/Q type Ca^+2^ channels and activate inflow rectifying K^+^ channels^18^. CB1 receptors are found extensively in the cerebrum, especially in the cerebral cortex, hippocampus, basal ganglia, and cerebellum^19^. Studies so far have shown that cannabinoids exert their behavioral and neuronal effects through cerebrum CB1 receptor activation^20^. On the other hand, cannabinoids have been known to exert anticonvulsant effects through CB1 receptors^2,3,21,22^. In the epileptic model created with pentylenetetrazole, ACEA was found to have an antiepileptic effect^9^ and inhibited the proconvulsant effect of toxoplasmosis^22^.

Synthetic cannabinoids are full agonists that bind to CB1 and CB2 receptors with a higher potency and affinity^23^. Since cannabinoids are known to exert their anticonvulsant effects on the CB1 receptor, selective CB1 receptor agonists and antagonists were used in the present study. For this reason, ACEA was preferred as the CB1 agonist and AM251 as the antagonist. Systemically administered cannabinoids undergo various pharmacokinetic interactions until they reach the cerebrum^20^. Therefore, we preferred to administer cannabinoids by i.c.v. route. Di Giacomo et al. showed that cannabidiol and cannabigerol have neuroprotective and antioxidant effects in rat astrocytes and isolated cortexes^24^. Marsicano et al. showed that cannabinoids have strong antioxidant effects in cerebellar granule cell cultures by inhibiting cell excitability with increasing K^+^ permeability and decreasing Ca^+2^ permeability through the CB1 receptor^25^.

In the present study, ACEA administration to the penicillin-injected rats decreased the MDA levels and increased SOD and GPx levels in the cerebrum and cerebellum tissues compared with the penicillin group. When AM251 was administered after penicillin, AM251 enhanced the activity of penicillin on MDA, SOD and GPx levels. Based on the data obtained, it is thought that ACEA, at a dose of 7.5 μg, acts on CB1 receptors presynaptically and reduces intracellular Ca^+2^ levels, preventing oxidative stress and thus suppressing the epileptiform activity. Thus, AM251 is thought to have the opposite effect of ACEA by increasing the intracellular Ca^+2^ level, increasing oxidative stress, and thus increasing the epileptiform activity induced by penicillin.

Recent studies have suggested that there may be a relationship between leptin and cannabinoids ^10-12^. Co-administration of ACEA and leptin significantly increased oxidative stress compared with the alone ACEA after penicillin injection. So, it was concluded that leptin reversed the effect of ACEA on oxidative stress. Combined administration of AM251 and leptin on our epileptic model no significant change was found in terms of oxidative stress compared to the AM251-administered group. From a pharmacological point of view, AM251 and leptin, which have proconvulsant effects when administered separately, are expected to significantly enhance oxidative stress when administered together. However, no significant differences were found. We suggested that AM251 and leptin have identical effects on oxidative stress by using similar pathways on Ca^+2^. Although our study revealed that leptin and cannabinoids exert their effects on epilepsy through the oxidative system, further molecular and biochemical studies are needed to further elucidate the molecular mechanisms. Findings underlying these interactions may lead to the development of new therapeutic strategies for the treatment of epilepsy.

## Data Availability

Data are available from the corresponding author on a reasonable account.
